# Leaving gift-giving behind: the ethical status of the human body and transplant medicine

**DOI:** 10.1007/s11019-018-9862-x

**Published:** 2018-08-13

**Authors:** Paweł Łuków

**Affiliations:** Instytut Filozofii, Uniwersytet Warszawski, Krakowskie Przedmieście 3, 00-927 Warsaw, Poland

**Keywords:** Transplant ethics, Human embodiment, Human person, Organ procurement, Sharing

## Abstract

The paper argues that the idea of gift-giving and its associated imagery, which has been founding the ethics of organ transplants since the time of the first successful transplants, should be abandoned because it cannot effectively block arguments for (regulated) markets in human body parts. The imagery suggests that human bodies or their parts are transferable objects which belong to individuals. Such imagery is, however, neither a self-evident nor anthropologically unproblematic construal of the relation between a human being and their body. The paper proposes an alternative conceptualization of that relation, the identity view according to which a human being is identical with their living body. This view, which offers a new ethical perspective on some central concepts of transplant medicine and its ethical and legal standards and institutions, supports widely shared intuitive ethical judgments. On this proposal, an act of selling a human body or one of its parts is an act of trade in human beings, not in owned objects. Transfers of human body parts for treatment purposes are to be seen as sharing in another human being’s misfortune rather than as giving owned objects. From the perspective of policy-making, the proposal requires, first, that informed consent for removal of transplant material be obtained from the potential benefactor. Secondly, explicit consent by the prospective benefactor is obligatory in the case of removal of transplant material from a living benefactor. Thirdly, in the case of posthumous retrieval, informed consent by the potential benefactor during their life is not ethically indispensable. Additionally, while refusal of posthumous retrieval expressed by a potential benefactor during their life must be respected, such a refusal needs ethical justification and explanation.

## Introduction

Metaphors and imageries matter. They shape the way we see things and the way we think how things should be (Lakoff and Johnson 2003). Reliance on a fitting metaphor or imagery may encourage the moral potential of individuals and societies. But a less than fitting metaphor or imagery can trigger difficulties which not only complicate thought but also trouble action.

The idea of gift-giving or donation and associated imagery has been the foundation of the ethics of organ transplants since the time of the first successful transplants in the 1950s (Hamilton 2012). The idea guides thinking about transfers of transplant material towards such ideals as selflessness, caring, and solidarity. Since these ideals imply condemnation of bodily transfers which involve profiting, *gift*-giving seems to provide a fitting conceptual framework and images. The idea of a gift also suggests condemnation of trafficking in human body parts, which has been gaining growing support in international and national bioethical regulations. Typical arguments against bodily exchanges for profit hold that such transactions are cases of or lead to—variously construed (Radin 1987; Nussbaum 1995; Joralemon and Cox 2003; Sharp 2000; Dickenson 2007)—objectification, commodification and commercialisation of the human body or its parts. By contrast, gift-giving requires transfers not to be for profit of any material kind.

There is, however, another side to the gift-giving imagery. Since it relies on the idea of transferable objects, it suggests the view of the relationship between an individual and his body as that of ownership or quasi-ownership. This idea, in turn, encourages moral views which contain the potential of objectification, commodification and commercialisation of the human body or its parts in various areas of social life. In the field of transplant medicine, this imagery can motivate not only selfless giving and condemnation of trade in human body parts, as found in bioethical regulations and guidelines (United States 1987; International Summit on Transplant Tourism and Organ Trafficking 2008; World Health Organization 2010; Nuffield Council on Bioethics 2011), but also (selfish) sale, more and more often proposed in response to scarcity of transplant material (Radcliffe-Richards et al. 1998; Boyer and Randall 2012; Gill and Sade 2002; Friedlaender 2002; Harvey 1990; Kishore 2005).

In order to block this potential of “dual use” of the imagery of the body-as-property, and its consequences that contradict important intuitive moral judgments and ethical and legal standards, I shall argue in the first section of this paper that the idea of gift-giving and its underlying imagery of the body-as-property should be abandoned. While the imagery is not self-contradictory, it is neither a self-explanatory nor a natural, nor an anthropologically unproblematic construal of the relation between a human being and his body. The imagery of the body-as-property does not originate in any uncontroversial facts about human beings, persons, or the universe. What is more important, since the institution of property is highly conventional, the imagery is potentially replaceable. Accordingly, there is enough conceptual space to replace the imagery of the human body as property or quasi-property by a view of human beings and their bodies, which responds more adequately to the dominant moral beliefs, which include condemnation of trade in human body or its parts.

In order to offer a more plausible and less arbitrary alternative to the above imagery, in the second section of this article, I will put forward a conceptualization of the human being, who—depending on her current biological or health status—can be a person, as identical with her living body. I will not argue extensively for this “identity view” of the relationship between a human being and their body. Relying mainly on other authors’ work, I will offer reasons for viewing human beings as identical with their living bodies in order to present a new ethical perspective on some central concepts of transplant medicine and its ethical and legal standards and institutions.

Most fundamentally, the identity view avoids risks of objectification, commodification and commercialisation of the human body in a more radical, because conceptual, way than it is possible within the frameworks that see the human body as an object of a (limited) ownership or quasi-ownership right. On this alternative proposal, selling the human body or its parts is an act of trade in *human beings* or their parts, not simply in their property. This re-conceptualization helps explain the condemnation of trade in the human body or its parts by seeing transfers of human body parts for treatment purposes not as a sharing of owned objects but as involved in the relationship of sharing in another human being’s misfortune. Sharing in another’s misfortune by making a body part available for transplant into their body, in which the transferred body part is a vector or carrier of assistance, is an entirely different relationship from sharing an owned object (e.g. in a commercial transaction). While sharing in another’s misfortune arises from concern for, and solidarity with, the less fortunate human being, a transfer of an owned object is at best a technical manoeuvre or a hands-off legal operation.

The identity view has also important policy and ethical consequences. First, like in the case of the body-as-property view, informed consent by the potential source to removal of transplant material is a necessary condition of the ethical legitimacy of such removal and subsequent transfer. However, such consent is not an exercise of a property or property-like right. It is mandatory because, and to the extent that, the procedure involves parties with their individual or personal rights. Secondly, explicit consent by the prospective source of transplant material is necessary in the case of removal of transplant material from a living human being, whereas in the case of posthumous retrieval, prior informed consent by the potential source of transplant material can be, within certain constraints, optional. Thirdly, while refusal of posthumous retrieval expressed by a potential source during her life must be respected, the potential source has a *prima facie* ethical obligation to justify and explain that refusal.

The analyses and proposals to be made below are conceptual, and have two main goals. The first is to identify the relations between the concept of ownership of one’s own body and some central ethical and legal norms that currently govern transfers of transplant material. To this extent, the analysis diagnoses ethical ramifications of a particular imagery. The second goal is to explore the ethical and policy implications of an alternative conceptual approach to transplant medicine.

## Ownership of the human body and human beings

The gift metaphor reigns over the ethics of transplant medicine. Organs and tissues are commonly said to be given or donated to recipients, which invokes powerful moral ideas of solidarity, caring, sharing, altruism, and sacrifice (Titmuss 1970). In this way, human body parts form a domain of circulation in which they are viewed as different or separate (or detachable) from individuals, moved, transferred, taken, inserted. They also define a sphere of moral relations, where they become a medium of altruism and generosity, which stem from commitment to the highest moral values, something which calls for appreciation and gratitude. The domain of selfless giving of such invaluable gifts typically relies on a relation of exclusive control by a human being over their body, the body being conceived of as property or quasi-property of that individual (to be referred to as “body-as-property”). This way of looking at the relation between a human being and their body can be found not only in ethical and medical literature but also, in some form, in law. Although no legal system affords the human body the status of property, individuals are sometimes granted possessory rights in parts of their own bodies (Yearworth et al. 2009; Moore 1990).

The imagery of the body-as-property has its own potential in the discourse of individual rights when individuals are said to have the right to control the way their bodies are used and by whom. The notion of the right to control one’s own body fits the concept of informed consent to medical intervention and to donation of transplant material. Within informed consent, transplant material removal is conditional on prior consent of the potential donor, where consent is seen as an exercise of both the right to self-determination, or an expression of autonomy, and of a property-like right in the removed part. Use of transplant material, such as implantation of someone’s body part into a recipient, is conditional on the “owner’s” consent to this use.

In an unrestricted version, the body-as-property imagery has profound normative consequences (Beyleveld and Brownsword 2000; Campbell 1992). It implies, first, free use of the body-property. No use of one’s own body can be prohibited, if such a use does not cause harm to others or does not violate their rights. In this way, the imagery of the body-as-property contains the justificatory potential for making one’s own bodily material available for transplant on the condition of financial gain to the “owner”. Secondly, it guarantees exclusive use of the body-property. Within the bounds of respect for the rights and protections of others against harm, no one is allowed to use the owner’s body without her explicit consent which determines the kind, duration and scope of that use. Retrieval or use of cadaver body parts is permissible on the basis of the explicit informed decision of the deceased person during her life, analogous to the disposal of the property by the owner in her last will. Thirdly, refusal to allow a use of the body, during the “owner’s” life or after his death, does not need special justification. Even if refusal to a use of one’s own body by others were to lead to their serious losses or harm the “owner” is justified in refusing. Someone who refuses to donate a part of his body for transplant, even in a case of the potential recipient’s dire need and with no serious risks to the “owner”, is in principle (with the exception of special relations between donors and recipients such as familial ties) beyond moral criticism.

The body-as-property conceptualisation suggests empowerment of individuals who are given dominion over their bodies. However, it seems to provide little or no ground for obligations towards others with regard to their bodies. This is because property is defined in terms of rights, which allow for derivations of (negative) duties to others and their bodies to the extent to which the right-holder’s rights are threatened. For example, I must not disfigure your body because this would constitute a violation of your property or property-like right in your body.

While the unrestricted version of the imagery of the body-as-property supports individual empowerment, it also has important ethical consequences. First, it seems to oppose some widely shared beliefs regarding duties of assistance to others, as for example the duty to help those who are sick. If my body is my property my help to an ill human being—by offering her a part of my body for transplant—can be an act of compassion or charity, which I am allowed to refuse to exercise with regard to this particular individual. My help is not (morally) owed her, unless I am specially related to her, e.g. emotionally, by family, or in some other way. My offering of my body part is a quasi-supererogatory act. In order to ground a strong (moral) duty of commission to assist the sick, the interventions performed on a potential donor’s body would have to issue from a different kind of moral basis than the right to free disposal of one’s property or quasi-property.

Secondly, the body-as-property imagery presupposes a conceptual disconnection of the human body or its parts from its “owner” (Leder 1999). It represents a human being as if there were two entities instead of one human being: a human being *and* her body. In effect, the imagery allows for seeing the human body as an “instrument” that belongs to someone. These disconnections may suggest that human bodies or their parts are objects like others. Seeing human bodies or their parts as objects, can in turn give rise to the normative proposals which contradict such widely supported ethical standards as the prohibition of the sale of human body parts, including one’s own.

The conceptualisation of the human body or its parts as an object would also contradict key international regulations that prohibit profiting from the human body or its parts. For example, Principle 6 of the Declaration of Istanbul provides that “Organ trafficking and transplant tourism violate the principles of equity, justice and respect for human dignity and should be prohibited” (International Summit on Transplant Tourism and Organ Trafficking 2008). According to article 21 of the Convention for the Protection of Human Rights and Dignity of the Human Being with regard to the Application of Biology and Medicine: Convention on Human Rights and Biomedicine, 1997 “The human body and its parts shall not, as such, give rise to financial gain.” (Council of Europe 1997a) and the Explanatory Report to the Convention states that financial gain from the human body or its parts “for the person from whom they have been removed or for a third party” is “an affront to human dignity” (131, 2) (Council of Europe 1997b). The Additional Protocol to the Convention on Human Rights and Biomedicine concerning Transplantation of Organs and Tissues of Human Origin, 2002 states in article 21 that “The human body and its parts shall not, as such, give rise to financial gain or comparable advantage.” and article 22 provides that “Organ and tissue trafficking shall be prohibited.” (Council of Europe 2002a). The Explanatory Report to this protocol also holds that “organs and tissues should not be bought or sold or give rise to direct financial gain for the person from whom they have been removed for a third party. Nor should the person from whom they have been removed, or a third party, gain any other advantage whatsoever comparable to a financial gain such as benefits in kind or promotion for example” (113), and that “Any trade in organs and tissues for direct or indirect financial gain … is prohibited” (119) (Council of Europe 2002b) (cf. also (Council of Europe 2015)).

Thirdly, the unrestricted version of the imagery of the body-as-property excludes an opt out regulatory framework for organ or tissue procurement that permits retrieval of cadaver body parts and their use in a transplant if the deceased person did not object to the retrieval and use during her lifetime. Such frameworks are quite common in Europe and elsewhere, and seem to become more widely accepted, as the recent examples of Wales and Scotland suggest. According to the imagery of the body-as-property, such frameworks lack moral legitimacy, even though opt out legislations are believed to (moderately) increase the numbers of deceased donors (Palmer 2012; Shepherd et al. 2014). Although acceptance for opt out systems is not as wide as the prohibition of financial gain from human bodily parts, the body-as-property idea does oppose the legal opt out regulations.

The provisions mentioned above clearly limit the rights of disposal of one’s own body parts during life or after death. They can be interpreted as rejecting the imagery of the body-as-property or as placing limits on a property right in the body. Accordingly, either human beings have a property right in their own bodies, which is limited by considerations different from harm to others or violation of others’ rights, or those individuals have non-property rights in their own bodies, and these rights limit acceptable uses of their own bodies, not only of the bodies of others. Since the provisions mentioned above are supported, among others, by references to protection of human dignity, one might speculate that they do not endorse the idea of property in one’s own body.

Despite the pervasiveness of the thinking about the human body as property, whether literally or metaphorically, there are serious reasons that speak against such conceptualisation. First, although found in so many cultures, property is a highly conventional idea. The institution of property regulates uses and allocation of goods when the potential demand for them exceeds supply. The institution of property is heterogeneous and multifaceted. What can be owned depends on often contingent particularities of social and political arrangements rather than on “the nature of things”. Historically, various kinds of entities were owned, including human beings in social systems based on slavery (Harris 1996). The specific forms and limits of property depend crucially on the particularities of normative systems to which they belong (Ypi 2011). In order to own something—as opposed to holding it or having physical control over it—one needs to live in a complex of normative relations to others and, perhaps, things. To say that something is mine, I must have a right to control it and to control others’ access to and use of it. Depending on the kind of object, ownership is composed of various “bundles of rights” and subject to different limits on their exercise (Honoré 1961), e.g. chattel property, real estate, or intellectual property. If therefore the human body or its parts are property it is the result of a collective decision of a society rather than a natural state of affairs, as libertarians seem to believe (Nozick 1974). Human bodies can be owned if societies, by means of their social and legal arrangements, choose to view them as such.

Secondly, such a decision not only contradicts the moral judgments articulated in the regulations mentioned above, but is also philosophically inadequate because it makes it difficult to make sense of the relation between human beings and their bodies. If human beings own their bodies it is not clear who or what these beings are. The imagery of the body-as-property suggests that human beings are somehow prior to or existentially independent of the property-rights they have in their bodies (Cohen 1995). Accordingly, the relationship between a human being and her body looks contingent in a way akin to the way in which the Cartesian soul or mind inhabits the human body. On this view, the human body is one among many possible material realisations of the human being. Thus, the body-as-property view implies that it is possible for a human being to survive *qua* a human being in a non-human body or perhaps without a body at all. While this idea is not self-contradictory—disembodied persons, ghosts, demons, and gods populate many folklores and, ironically, significant and prominent philosophical literature—it is highly questionable when applied to humans: What is human about a human being without a human body?

Since property is a highly conventional institution and the relation between a human being and his body is not contingent in the way in which the relation between an owner and the object owned is, the conceptualisation of the relationship between a human being and his body in terms of property is not as straightforward or elementary as the imagery of the body-as-owned may suggest. The imagery presents the relation as conventional and contingent, and so it is a questionable foundation for an adequate normative view.

## The human being and the human body

A particularly helpful insight into the non-contingent nature of the relation between a human being and her body is provided by the phenomenological distinction between a body (*Körper*) and the lived body (*Leib*) (Husserl 1970). It acknowledges the centrality of the body in human experience not only from the perspective of the uses one can make of one’s own body but in particular from the point of view of the role it plays in individual experience and identity (Hanna and Thompson 2003). The concept of the lived body exposes the obvious fact that to the extent to which the biological structure of the human organism determines the ways in which human beings interact with the natural and social environments, the human body is an indispensable and constitutive component of every such being.

Humans communicate with their environment and with one another through bodily or sense perception. Their identity and sense of identity are constituted by perceptions of what is not them (the environment) and of their own bodies (e.g. interoception and proprioception). They are what they are as the outcome of interactions between their living bodies and the environment and as the active unit of those interactions which single out a particular body as an individual (Merleau-Ponty 1962). Human existential experiences (like suffering, fear, joy, love) are also inseparable from human embodiment (Heidegger et al. 2010).

The experiential centrality of the human body in human identity formation indicates a non-contingent nature of the relationship between a human being and its body in that the body is necessary for the formation of identity of a human being *qua* human being and as a particular human being. One might interpret this necessity causally and say that the functions of a living human body cause species identity and the identity of an individual member of that species. This view, however, implies a duality between a particular being and its bodily “basis”. While this view could see the relationship between a human being and its body as non-contingent, it would also imply an unacceptable thesis, according to which there are two entities involved in the existence of a human being: a living body (the cause) and its effect, the human being itself.

A more preferable interpretation of the non-contingent role of the body in both the species and individual identity of a human being is numerical identity. A living human body is necessary for the identity of a human being because a particular human being (an individual member of *Homo sapiens*) is strictly numerically identical with a particular living human body (van Inwagen 1980; Snowdon 1990; Olson 1997; Blatti and Snowdon 2016). The biological processes within that body, which are determined by the biological makeup of the *Homo sapiens* (species identity), sustain the experiences and interactions between that human being, its environment and itself, and in this way they play a central role in the emergence of the unique identity of a particular human being. During standard biological development, a particular human being, identical with a particular living human body, matures into a being who, due to her psychological capacities and interactions with others, functions as a human person or an active member of a moral community. Illness, infirmity or senescence can change the social status of a human being as a person but they do not change her identity as a human being. Therefore, there can exist human beings who are not human persons yet (such as human foetuses) and human beings who ceased to be human persons (such as those in a persistent vegetative state). There also exist dead human bodies which are not human persons any more.

The identification of a human being with his or her living body does not preclude some changes in the make-up of a particular human being. It is possible for a human being to have a body part added or detached without questioning that individual’s continuity as a particular human being. Growth, especially at the earlier stages of ontogeny, is a natural process during which new body parts are formed; replacement of skin cells, loss of hair or nails, are standard, although not particularly significant, natural processes during which human beings change without losing their identity or integrity. Thus, a human being can maintain their identity and integrity when injured or mutilated in an accident or assault, when a body part is removed (e.g. for transplant) or when subjected to an intervention such as body modification or treatment (e.g. implantation of a body part surgically removed from another human being). The identity of a particular human being with their living human body does not imply that they remain totally unchanged throughout their entire life. Quite the reverse: no change at all would imply no life.

A human being *is* a living human body, which, as the result of normal biological development, usually becomes a person. Human bodies are not objects that mediate between human beings and their environment. Nor are human bodies Cartesian “containers” inhabited by beings who could reside in different containers or be transferred into different bodies, as the majority of the most influential work on personal identity assumes in various discussions of thought experiments involving transfers of the contents of the mind, such as uploads into computers (Strawson 1959; Shoemaker 1970; Nozick 1981; Parfit 1984; Noonan 1989; Unger 1990). Human beings and human persons with their unique identities are necessarily realised in the bodies of the *Homo sapiens* species. If a being could exist in a realisation significantly different from the body of the species *Homo sapiens* it would not be a human being (Williams 1973). Differently put, a human being without a human body is a non-entity (van Inwagen 1997).

From the experiential and ethical points of view, a living human body cannot therefore be viewed as an object like any other, and so as a candidate for property. Its special status is owed not to its association with or connection to a person, who happens to inhabit or own it, but to its constitutive role in the make-up of a human being and, consequently, of a human person i.e. for a sufficiently mature human being (Ricœur 1992; Mackenzie 2001). To ignore the living human body in conceptualisations of human beings, or to see it as an object singled out merely by its inseparability from a person rather than by its identity with a human being, is at best to ignore what makes a human being, and a human person, human.

The identity view provides a framework for more intuitive justifications of important ethical norms than the body-as-property view. In the present context, of special significance are prohibitions such as those against injury, coercion, enslavement, and murder. They are not to be seen as protections against assaults on someone’s property or quasi-property (their body), with no harm to the owner; nor are they to be viewed as assaults on persons who are in no special or non-contingent way interfered with by such actions. From the perspective of their nature, injury, coercion, enslavement, and murder are to be seen, first, as exactly what they are: attacks on individual human beings, not on someone’s property or quasi-property; as violations of rights of individuals (or personal rights legally understood), not as violations of rights in property. Secondly, the seriousness of such assaults lies in the fact that they damage the very entity and existence of human beings.

In parallel to the change of perspective on injury, coercion, enslavement, and murder, the identity view encourages an alternative picture of illness, disability, infirmity, and death. As before, they are not to be understood as malfunctions, damages to or destructions of anyone’s property, without immediate harm to the owner; nor as attacks on persons, who cannot be in any special way affected by such irregularities regarding their bodies. From the point of view of their nature, illness, disability, infirmity, and death are harm to human beings with their beliefs, desires, and value commitments. Illness, disability, and infirmity affect human beings in the most intimate ways. As literature on illness, disease, disability, infirmity, suffering, and pain, shows, they can transform human beings (psychologically and, occasionally, physically), in fundamental ways (Sacks 2006; Sontag 1990; Toombs and Kay 1992; Svenaeus 2011). They can modify an individual’s perception of reality and self-perception, rearrange their priorities and sensitivities, and various other aspects of their individuality as a human being. (Death changes and destroys a human being most radically and irrevocably into a corpse.) When these changes and modifications are experienced as evils, as they typically are, it is not only because they are inconveniences of some size or gravity but because they are threats to the very existence and identity of a human individual. They are at best disruptions of the continuity of the affairs of an individual, and at worst their annihilation. It is because of the most intimate relationship between a human being and their body that such assaults on the body as illness, disability, infirmity, and death can be evils.

The identity view sketched above presents the relationship between a human being and their living body as non-trivial and non-contingent. It also allows for an interpretation of harm caused by illness or disability as sufficiently serious to invite moral response in the form of assistance. Depending on the nature of the harm and the abilities of those who recognise it in a particular case, assistance can take various forms. It can be psychological support, medical treatment, or—as it is the case in transplants—transfer of a body part from one human being into the body of another human being. Since on the identity view such a transfer is not a transfer of property or quasi-property, it reconceptualises the ethics of transplants.

## An alternative perspective on the ethics of transplant medicine

Once one rejects the distinction between a human being and her owned body in favour of appreciation of human embodiment, the imagery of transplant medicine changes dramatically. This change of the very ethical paradigm of transplant medicine becomes visible from the perspective of the four difficulties that are generated by the imagery of body-as-property and which were discussed earlier in the first section.

First, the substitution of the conceptualisation of body-as-property by the identity view blocks proposals for permissibility of sale of a human body or its parts by abandoning the conceptual space which allows for sale talk. Once a living human body is seen as identical with human being, the decision concerning her body or its parts is a decision made by that being about *herself*, not about an object which is different from her. In this way, proposals to permit commerce in human bodies or body parts lack a key conceptual resource. The human body is not even a candidate for something that can be owned. Accordingly, even though its parts can be transferred from one human being to another, it is not a possible unit of *property* transfers. Objectification, commodification or commercialisation of the human body or its parts become conceptually impossible because human bodies are not conceptually separate from human beings with their rights. The decision maker with her individual or personal rights is not split into two entities, one of which is a transferable object of property rights that pertain to the other.

Secondly, the conceptualisation of the human body as identical with a human being changes the semantics of transplant ethics, and so it transforms its normative framework. In the vocabulary of body-as-property, a decision to *donate* or give a body part for transplant is an exercise of a property or property-like right. In the account of the identity of human beings and their bodies, a decision to allow others to remove a body part in order for it to be transferred into someone else is an exercise of a personal right such as, among others, the right to self-determination or privacy. Obviously, personal rights are exercised during their holders’ lives. What is being decided upon by exercises of those rights, can, however, materialise during the lives of their holders (“live donation”) or after their death (“posthumous donation”). The key difference between someone’s decision to permit removal of his body part and its transfer into someone else during his life (“live donation”) and his decision to permit such a removal and transfer after his death (“posthumous donation”) lies in the timing of execution of what has been decided upon. In the case of the so-called live donation it occurs while the donor is still a person, whereas in the case of the so-called posthumous donation the transfer takes place at the time when the donor is not a person any more. In this respect the exercise of a personal right does not differ fundamentally from exercise of property- or property-like rights. However, the nature of the right exercised in such decisions differs fundamentally from the nature of a property- or property-like right.

Since on the identity view bodies or their parts cannot be property, the moral nature of transplants is reconceived. Decisions to transfer one’s own body part into someone else in need are not to be seen as acts of *giving*. Instead, they should be seen as *sharing in the misfortune* of another person. Sharing in someone else’s misfortune is an act of beneficence which arises from recognition of that individual’s actual or future need and from the current caring about them. The result of that concern may be a decision to consent to transfer of one’s own body part into someone else during the decision maker’s life or after their death. The transfer is not understood as an act of giving a body part but as a result of a decision to initiate an interaction with others, some of whom are assisted during the decision maker’s life and others after his death. The interaction begins in the present but its results may occur after the decision maker’s death, analogously to the consequences of making promises which are directed towards the future. From an ethical point of view, transplant is therefore not a transfer of an owned body part from the *donor* to its *recipient* or sharing that part with the recipient—as some valuable un-orthodox views of transplants propose (Sharp 2016)—but a vehicle or vector of a *person’s sharing in* the lives of the parties to the transfer of bodily material. A decision to transfer one’s own body part into someone else’s body is therefore an exercise of an individual or personal right by personal engagement with the other human being.

Sharing in another’s life is a sequence of events. The decision to transfer a body part from one human being to another is the first event in the sequence, whereas the transfer itself may be a part of that sequence or not. In contrast with the standard idea of gift-giving, sharing in someone else’s misfortune is not an event but a time-extended process. In the case of the decision to transfer a bodily part during the benefactor’s life it usually continues (or is intended to continue) after the transfer of the bodily part, which makes it akin to the classical anthropological accounts of gift-giving. In many societies transfers are forms of an on-going process of involvement and communication building and maintenance, which are embedded in society’s life, rather than merely an act of exchange for (material or of other nature) profit performed by isolated and self-sufficient individuals (Mauss 1990; Lévi-Strauss 1969). In the case of the decision to transfer a body part after the benefactor’s death, the process of sharing in others’ misfortunes is taking place during his life.

The identity approach is not only more adequate anthropologically than the gift-giving imagery but it can also support a more plausible view of the ethics of organ transplant by being adapted to the nature of human social interactions. The current standards, which require that benefactors (or their surviving relatives) and beneficiaries remain anonymous to each other unless medically necessary (World Health Organization 2010, Principle 11; Council of Europe 2002a, art. 23), prevent actual social interaction between the beneficiary and the benefactor. By seeing transplant as a one-time event, they are not responsive to the need of (continuation of) the social relation between the benefactor and the beneficiary. This need, however, becomes prominent when surviving family members of the benefactor ponder the future of the organ removed from a deceased loved one or when beneficiaries inquire about their benefactors. By contrast, transplant ethics based on sharing in another person’s misfortune recognises this need because it sees transfer of a body part as a time-extended process with its normal stages and succession of events, which require suitable conclusion on psychological, social, and moral levels. Within appropriate limits and safeguards, an ethics of transplant medicine based on the identity view makes non-anonymity of benefactors and beneficiaries in some cases permissible. Such an ethics can help reduce the “tyranny of the gift” (Fox and Swazey 1992) to the extent to which it stems from the absence of social interaction between benefactors and beneficiaries.

Thirdly, since the identity view understands decisions to transfer one’s own body parts as exercises of personal rights with regard to oneself, consent to bodily material removal and transfer appears in a new light. To the extent to which individuals are rights holders, decisions to donate made by living human beings are indispensable for legitimate removal of transplant material from them. The decision to donate is a precondition of removal at least as strongly as in the body-as-property account. A fundamental change occurs however as regards the nature of such consent, because a different kind of right (i.e. a personal right) is involved, and so its legitimising role and scope is different. Consequently, the regulatory framework based on the identity approach to consent to bodily material removal and transfer might need to be changed.

On the identity view, removal of a bodily part from a living person without explicit consent of the potential benefactor is unacceptable as long as it is assumed that human beings have liberty rights, including the right to self-determination. Since on this approach, a deceased human being is not a person any more, retrieval of bodily parts from human cadavers without consent of the dead individual obtained during her life can be legitimate. It does not violate the personal rights of that person because she is non-existent now. Of course, as suggested by the earlier discussion of live and posthumous removal and transfer of bodily material, other issues can emerge, and they can limit the form or range of use of a dead human body. Taking care of her posthumous interests (Feinberg 1993; Pitcher 1993; Wilkinson 2011, Chap. 3 & 4), a deceased human being could have decided during her life that no part of her corpse be removed for the purpose of transplant or for any other goal, or she could express special wishes concerning *post mortem* uses of her dead body (e.g. for research purposes). Members of her family may have familial concerns regarding the disposal of her dead body; state interests might also be involved, etc. However, retrieval of transplant material from a human corpse does not in principle require the deceased person’s approval during her lifetime unless and until she had made rightful decisions regarding her dead body. To the extent to which there are no serious reasons that demand prior explicit decisions regarding the use of someone’s body after her death (such as posthumous interests, rights and entitlements of the living others, or interests of the state), the reconceptualization offered above permits opt out regulatory frameworks for retrieval of cadaveric transplant material without rejecting the alternative of opt in frameworks.

The fact that a dead human body is not a person any more does not necessarily imply that anyone who happens to physically control that body earns property rights in it. Death of a human being does not, of itself, turn the remaining body into potential or actual property. Its status seems to escape the distinction between things and persons, and various normative restrictions and requirements can apply to the surviving members of a society with reference to the handling of that dead body. Apart from carrying out decisions made by the deceased during his life, the entitlements of surviving relatives or state interests etc., we can see a dead body as a symbol or a memento of the deceased. From this perspective, various restrictions can govern what can be done to or with it, as for example those relating to the memory of the person who passed away, the feelings and reminiscences of those left behind, mourning etc. In the manner in which a person’s fame during life commands respect and protection by the living, the public remembrance of a deceased person may be an object of decisions of both the dead human being during his life and by those remaining behind after that person’s death.

Fourth, unlike the body-as-property view, the identity approach suggests that refusal to offer parts of one’s own body for transplant involves a *prima facie* moral obligation to justify the decision. Since the existence and identity of a human being depends on others (Ruddick 1989; Kittay 1999; MacIntyre 1999), human beings have a *prima facie* moral duty to support others or helpfully share in their misfortunes. The obligation can be supported by, among others, compassion, empathy, or reciprocity. Its strength and the extent of provision required can vary, depending on the emotional proximity of the potential beneficiary of a bodily transfer to the prospective benefactor, or the health status of the prospective beneficiary and the urgency of their need, in the case of removal of a body part from a living human being. The *prima facie* moral obligation to make one’s bodily material available for removal and transfer into the body of a person in need of treatment can be stronger in the case of close relatives and weaker vis-à-vis strangers. Human societies recognise that their members ought to bear certain burdens for the sake of their loved ones. The strength of the moral obligation to justify refusal can also depend on how large a burden the potential benefactor would have to accept to share in another person’s misfortune. In the case of retrieval and transfer of bodily parts after the benefactor’s death, the burden does not exist, and so refusal of consent to posthumous retrieval and transfer would usually require relatively strong reasons (of moral, religious, or emotional nature). If, however, removal of transplant material implied significant burden to the potential benefactor, as it is possible in the case of some transplants from living benefactors, refusal would require little or no justification.

## Conclusion

It has been argued that the gift-giving metaphor associated with transplant ethics, albeit helpful in many respects, is based on the perception of the human body as an object of more or less exclusive control, which is akin to property. One of the consequences of such conceptualisation of the human body is that it opens the possibility of, or perhaps encourages, objectification, commodification and commercialisation of the human body and its parts. In the field of transplant medicine, it supports proposals to introduce trade in human body parts, which contradicts widely held moral beliefs and national and international regulations. The view of the human body as property is philosophically inadequate to the extent to which it is based on a questionable view of the human being; it is also anthropologically flawed, as it does not recognise the importance of exchanges which are not for gain. A more acceptable view of the relationship between the human being and the human body relies on the concept of identity. Although full development of such a view requires further studies (some of which are already available in the literature), it is clear that such a view can help block the idea of property-like rights in the human body, and so it contains conceptual resources to forestall objectification, commodification and commercialisation of the human body or its parts.

In the field of transplant medicine such a construal can discourage proposals for bodily exchanges for profit, without surrendering the ideals of selflessness, caring and solidarity. It offers intuitively more adequate approaches to transplant medicine and its ethics by viewing transplants as part of a larger project of solidarity grounded in sharing in each other’s lives. By providing a conceptual framework for opt out regulatory solutions regarding transplant material procurement, the identity view can also potentially increase the number of lives saved.

## References

[CR1] Beyleveld Deryck, Brownsword Roger (2000). My Body, My Body Parts, My Property?. Health Care Analysis.

[CR2] Blatti Stephan, Snowdon Paul F (2016). Animalism: New Essays on Persons, Animals, and Identity.

[CR3] Boyer J, Randall (2012). Gifts of the Heart… and Other Tissues: Legalizing the Sale of Human Organs and Tissues. Brigham Young University Law Review.

[CR4] Campbell CS (1992). Body, Self, and the Property Paradigm. Hastings Center Report.

[CR5] Cohen GA (1995). Self-Ownership, Freedom, and Equality.

[CR6] Council of Europe (1997). Convention for Protection of Human Rights and Dignity of the Human Being with Regard to the Application of Biology and Biomedicine: Convention of Human Rights and Biomedicine (CETS No. 164).

[CR7] Council of Europe (1997). Explanatory Report to the Convention for the Protection of HUMAN Rights and Dignity of the Human Being with Regard to the Application of Biology and Medicine: Convention on Human Rights and Biomedicine.

[CR8] Council of Europe (2002). Additional Protocol to the Convention on Human Rights and Biomedicine concerning Transplantation of Organs and Tissues of Human Origin (CETS No. 186).

[CR9] Council of Europe (2002). Explanatory Report to the Additional Protocol to the Convention on Human Rights and Biomedicine concerning Transplantation of Organs and Tissues of Human Origin.

[CR10] Council of Europe, Steering Committee on Bioethics (2015). Convention against Trafficking in Human Organs (CETS No. 216).

[CR11] Dickenson Donna (2007). Property in the Body: Feminist Perspectives. Cambridge Law, Medicine, and Ethics.

[CR12] Feinberg Joel, John Martin Fischer (1993). Harm to Others. The Metaphysics of Death.

[CR13] Fox Renee C, Swazey Judith P (1992). Spare Parts: Organ Replacement in American Society.

[CR14] Friedlaender MM (2002). The Right to Sell or Buy a Kidney: Are We Failing Our Patients?. Lancet.

[CR15] Gill Michael B, Sade Robert M (2002). Paying for Kidneys: The Case Against Prohibition. Kennedy Institute of Ethics Journal.

[CR16] Hamilton David (2012). A History of Organ Transplantation: Ancient Legends to Modern Practice.

[CR17] Hanna Robert, Thompson Evan (2003). The Mind-Body-Body Problem. Theoria Et Historia Scientiarum.

[CR18] Harris JW (1996). Who Owns My Body. Oxford Journal of Legal Studies.

[CR19] Harvey J (1990). Paying Organ Donors. Journal of Medical Ethics.

[CR20] Heidegger Martin, Joan Stambaugh, Schmidt Dennis J (2010). Being and Time. SUNY Series in Contemporary Continental Philosophy.

[CR21] Honoré AM, Guest AG (1961). Ownership. Oxford Essays in Jurisprudence: A Collaborative Work.

[CR22] Husserl Edmund (1970). The Crisis of European Sciences and Transcendental Phenomenology; An Introduction to Phenomenological Philosophy. Northwestern University Studies in Phenomenology & Existential Philosophy.

[CR23] International Summit on Transplant Tourism and Organ Trafficking (2008). The Declaration of Istanbul on Organ Trafficking and Transplant Tourism.

[CR25] Joralemon D, Cox P (2003). Body Values: The Case Against Compensating for Transplant Organs. Hastings Center Report.

[CR26] Kishore RR (2005). Human Organs, Scarcities, and Sale: Morality Revisited. Journal of Medical Ethics.

[CR27] Kittay Eva Feder (1999). Love’s Labor: Essays on Women, Equality, and Dependency. Thinking gender.

[CR28] Lakoff George, Johnson Mark (2003). Metaphors We Live by.

[CR29] Leder Drew, Cherry MJ (1999). Whose Body? What Body? The Metaphysics of Organ Transplantation. Persons and Their Bodies: Rights, Responsibilities, Relationships.

[CR30] Lévi-Strauss, Claude. 1969. *The Elementary Structures of Kinship*. Rev. Aufl. Boston: Beacon Press.

[CR31] MacIntyre Alasdair C (1999). Dependent Rational Animals: Why Human Beings Need the Virtues. The Paul Carus Lecture Series.

[CR32] Mackenzie Catriona, Kay Toombs (2001). On Bodily Autonomy. Handbook of Phenomenology and Medicine.

[CR33] Mauss, Marcel. 1990. *The Gift: The Form and Reason for Exchange in Archaic Societies*. (trans: Halls, W.D.). London: Routledge.

[CR34] Merleau-Ponty Maurice (1962). Phenomenology of Perception.

[CR35] Moore V. Regents of the University of California. July 9, 1990. Supreme Court of California.

[CR36] Noonan Harold W (1989). Personal Identity.

[CR37] Nozick Robert (1974). Anarchy, State, and Utopia.

[CR38] Nozick Robert (1981). Philosophical Explanations.

[CR39] Nuffield Council on Bioethics (2011). Human Bodies: Donation for Medicine and Research.

[CR40] Nussbaum Martha C (1995). Objectification. Philosophy and Public Affairs.

[CR41] Olson Eric T (1997). The Human Animal: Personal Identity Without Psychology. Philosophy of Mind Series.

[CR42] Palmer, Melissa. 2012. *Opt*-*Out Systems of Organ Donation: International Evidence Review*. Welsh Government Social Research. http://wales.gov.uk/docs/caecd/research/121203optoutorgandonationen.pdf. Accessed 12 Aug 2018.

[CR43] Parfit Derek (1984). Reasons and Persons.

[CR44] Pitcher George, John Martin Fischer (1993). The Misfortunes of the Dead. The Metaphysics of Death.

[CR45] Radcliffe-Richards J, Daar AS, Guttmann RD, Hoffenberg R, Kennedy I, Lock M, Sells RA, Tilney N (1998). The Case for Allowing Kidney Sales. International Forum for Transplant Ethics. Lancet.

[CR46] Radin Margaret J (1987). Market-Inalienability. Harvard Law Review.

[CR47] Ricœur Paul (1992). Oneself as Another.

[CR48] Ruddick Sara (1989). Maternal Thinking: Toward a Politics of Peace.

[CR49] Sacks, Oliver. 2006. *The Man Who Mistook His Wife for a Hat and Other Clinical Tales*. Touchstone Aufl. New York, NY: Simon & Schuster.

[CR50] Sharp Lesley A (2000). The Commodification of the Body and Its Parts. Annual Review of Anthropology.

[CR51] Sharp Lesley A, Erik Malmqvist, Zeiler Kristin (2016). Sharing Amidst Scarcity: The Commons as Innovative Transgression in Xeno- and Allo-transplant Science. Bodily Exchanges, Bioethics and Border Crossing: Perspectives on Giving, Selling and Sharing Bodies.

[CR52] Shepherd L, O’Carroll RE, Ferguson E (2014). An International Comparison of Deceased and Living Organ Donation/Transplant Rates in Opt-in and Opt-out Systems: A Panel Study. BMC Medicine.

[CR53] Shoemaker Sydney (1970). Persons and Their Pasts. American Philosophical Quarterly.

[CR54] Snowdon Paul F, Christopher Gill (1990). Persons, Animals, and Ourselves. The Person and the Human Mind: Issues in Ancient and Modern Philosophy.

[CR55] Sontag, Susan. 1990. *Illness as Metaphor; and, AIDS and Its Metaphors*. 1^st^ Anchor Books Aufl. New York: Doubleday.

[CR56] Strawson PF (1959). Individuals.

[CR57] Svenaeus F (2011). Illness as Unhomelike Being-in-the-World: Heidegger and the Phenomenology of Medicine. Medicine, Health Care and Philosophy.

[CR58] Titmuss Richard Morris (1970). The Gift Relationship: From Human Blood to Social Policy.

[CR59] Toombs S, Kay (1992). The Meaning of Illness: A Phenomenological Account of the Different Perspectives of Physician and Patient. Philosophy and Medicine.

[CR60] Unger Peter K (1990). Identity, Consciousness, and Value.

[CR61] United States. 1987. *Organ Transplantation: Issues and Recommendations: Report of the Task Force on Organ Transplantation. U.S*. Washington, DC: Department of Health and Human Services, Public Health Service, Health Resources and Services Administration, Office of Organ Transplantation.

[CR62] van Inwagen Peter, van Peter Inwagen (1980). Philosophers and the Words ‘Human Body’. Time and Cause.

[CR63] van Inwagen Peter (1997). Materialism and the Psychological-Continuity Account of Personal Identity. Philosophical Perspectives.

[CR64] Wilkinson TM (2011). Ethics and the Acquisition of Organs. Issues in Biomedical Ethics.

[CR65] Williams Bernard, Williams Bernard (1973). Personal identity and individuation. *Problems of the Self: Philosophical Papers
1956–1972*.

[CR66] World Health Organization. 2010. Guiding principles on human organ transplantation. As endorsed by the sixty-third World Health Assembly in May 2010. In *Resolution WHA63.22*, ed. World Health Organization. http://www.who.int/transplantation/Guiding_PrinciplesTransplantation_WHA63.22en.pdf. Accessed 12 Aug 2018.

[CR24] Yearworth and others v North Bristol NHS Trust 2009. EWCA Civ 37. http://www.bailii.org/cgibin/format.cgi?doc=/ew/cases/EWCA/Civ/2009/37.html&query=(yearworth). Accessed 12 Aug 2018.

[CR67] Ypi Lea (2011). Self-ownership and the State: A Democratic Critique. Ratio.

